# Parameterizing network graph heterogeneity using a modified Weibull distribution

**DOI:** 10.1007/s41109-023-00544-9

**Published:** 2023-04-28

**Authors:** Sinan A. Ozbay, Maximilian M. Nguyen

**Affiliations:** 1 Bendheim Center for Finance, Princeton University, Princeton, NJ USA; 2 Lewis-Sigler Institute, Princeton University, Princeton, NJ USA

## Abstract

We present a simple method to quantitatively capture the heterogeneity in the degree distribution of a network graph using a single parameter $\sigma$. Using an exponential transformation of the shape parameter of the Weibull distribution, this control parameter allows the degree distribution to be easily interpolated between highly symmetric and highly heterogeneous distributions on the unit interval. This parameterization of heterogeneity also recovers several other canonical distributions as intermediate special cases, including the Gaussian, Rayleigh, and exponential distributions. We then outline a general graph generation algorithm to produce graphs with a desired amount of heterogeneity. The utility of this formulation of a heterogeneity parameter is demonstrated with examples relating to epidemiological modeling and spectral analysis.

## Introduction

Many real-world processes and phenomena can be accurately characterized as instances of graphs or dynamic processes on graphs (Newman 2018). Recent attention to graph models have been made in the areas of statistical models (Cheng and Titterington 1994; Sarle 1994; Jordan 2004; Goldenberg 2010), social network analysis (Scott 1988), epidemiological models (Pastor-Satorras et al. 2015), political polarization models (Conover 2011), and several other domains. For certain modeling problems, while it would be ideal to determine all of the nodes and edges of the graph empirically, the ability to do so is often limited in practice and prone to error. Thus, it is common to estimate the global or local topological features of the graph and then generate a graph matching those features on which simulations and computations can be performed and conclusions can be extrapolated.

One key feature of graphs that is of interest is the heterogeneity of a graph, which captures the amount of structural symmetry the graph encodes. To illustrate this intuitively, imagine a complete graph with N nodes, such that each node is connected to all other $N - 1$ nodes. Any process on this network would be indifferent to its particular starting point as each node is equally connected to all other nodes. Such a graph is considered homogeneous in its connectivity. On the other extreme, a graph where there is much more variation in what a single node is connected to encodes much more local structure. These types of graphs display a large amount of heterogeneity. Many processes that can be modelled with and simulated on graphs, such as epidemics, wild fires, diffusion problems, etc. are known to be sensitive to the amount of heterogeneity present in the graph modelling the system. As a result, having a rigorous and quantitative notion of heterogeneity that can be measured and controlled in simulations and calculations would be useful.

While qualitative comparisons between the heterogeneity of different types of graphs is common, it is difficult to make precise quantitative comparisons of the heterogeneity of graphs that generalize to many graph types for several reasons. First, there are many different types of graphs. Canonical examples include random graphs such as the Erdos–Renyi model, power-law graphs, small-world networks, complete and regular graphs, bipartite graphs, multiplex graphs, and many more that are of interest for varying real-world applications. Second, many of these graphs are generated using entirely different graph construction procedures. In light of the variation in how these graphs are generated, producing a parameterization that would allow for a simple interpolation between all of these different graphs is a seemingly difficult task.

A number of approaches to capturing heterogeneity are discussed in the literature. These include using the degree variance as a heterogeneity index (Snijders 1981; Bell 1992; Smith and Escudero 2020), spectral approach (Von Collatz and Sinogowitz 1957), branching index (Estrada 2010), the Gini coefficient (Hu and Wang 2008), among others (Albertson 1997; Jacob 2016).

Furthermore, a number of approaches have been proposed that generate graphs of a given distribution which can recover certain distributions of interest. These methods continuously interpolate between graphs of various degree distributions by adjusting tunable parameters that control the attachment probabilities of a new node to existing nodes. Examples include the growing random network (GN) method (Krapivsky et al. 2000) and the inverse preferential attachment (IPA) model (Barabási et al. 1999; Liao and Hayashi 2022).

We note a particular approach that interpolates between two specific graphs, the Erdos–Renyi model and a scale-free network (Gómez-Gardeñes and Moreno 2006). This method allows for a parameterization of heterogeneity wherein the first graph is quite homogeneous in its degree distribution and the second is very heterogeneous. However, this interpolation method is limited in the two ways described above. First, this approach only allows for a comparison between two graphs at a time. Second, this method cannot necessarily be easily extended to consider other pairs of graphs since it interpolates at the level of graph construction, for which certain pairs of graphs would be strictly incompatible. However, as we will see below, by abstracting from the graphs themselves and instead focusing on the degree distributions, it becomes possible to quantitatively measure heterogeneity.

## Methods

To solve the above problem, we present a control parameter that quantitatively represents the amount of heterogeneity in a graph as measured by the heterogeneity in its degree distribution. As the motivating example in the introduction shows, the heterogeneity is tightly, though not exactly, tied to the amount of local structure embedded in a graph, with the degree of nodes encoding all of the first order (or first neighbor) information about any given node. Thus, we make the key assumption that the heterogeneity of the graph can be accurately captured and quantified as a function of the degree distribution of the graph.

Once we make this assumption, we notice that the degree distributions of many graphs of empirical interest are given as special cases of the Weibull distribution. Recall the probability density function of the 2-parameter Weibull distribution (Rinne 2008):

1 $$\begin{aligned} f(x; \lambda , \alpha ) = \frac{\alpha }{\lambda }\left( \frac{x}{\lambda }\right) ^{\alpha - 1} e^{-(x/\lambda )^\alpha }; x\ge 0; \alpha , \lambda > \mathbb {R}^+ \end{aligned}$$

where $\alpha$ is the shape parameter and $\lambda$ is the scale parameter. We note the following special cases of this function:

- $\alpha < 1$ corresponds to heavy-tailed degree distributions, with subexponential decay in the tail.
- $\alpha = 1$ recovers the exponential distribution.
- $\alpha = 2$ recovers the Rayleigh distribution.
- $\alpha = 3.4$ corresponds to an approximately Gaussian distribution, which can recover the Erdos–Renyi graph.
- $\alpha \rightarrow \infty$ recovers a $\lambda$-regular graph.

**Fig. 1:**
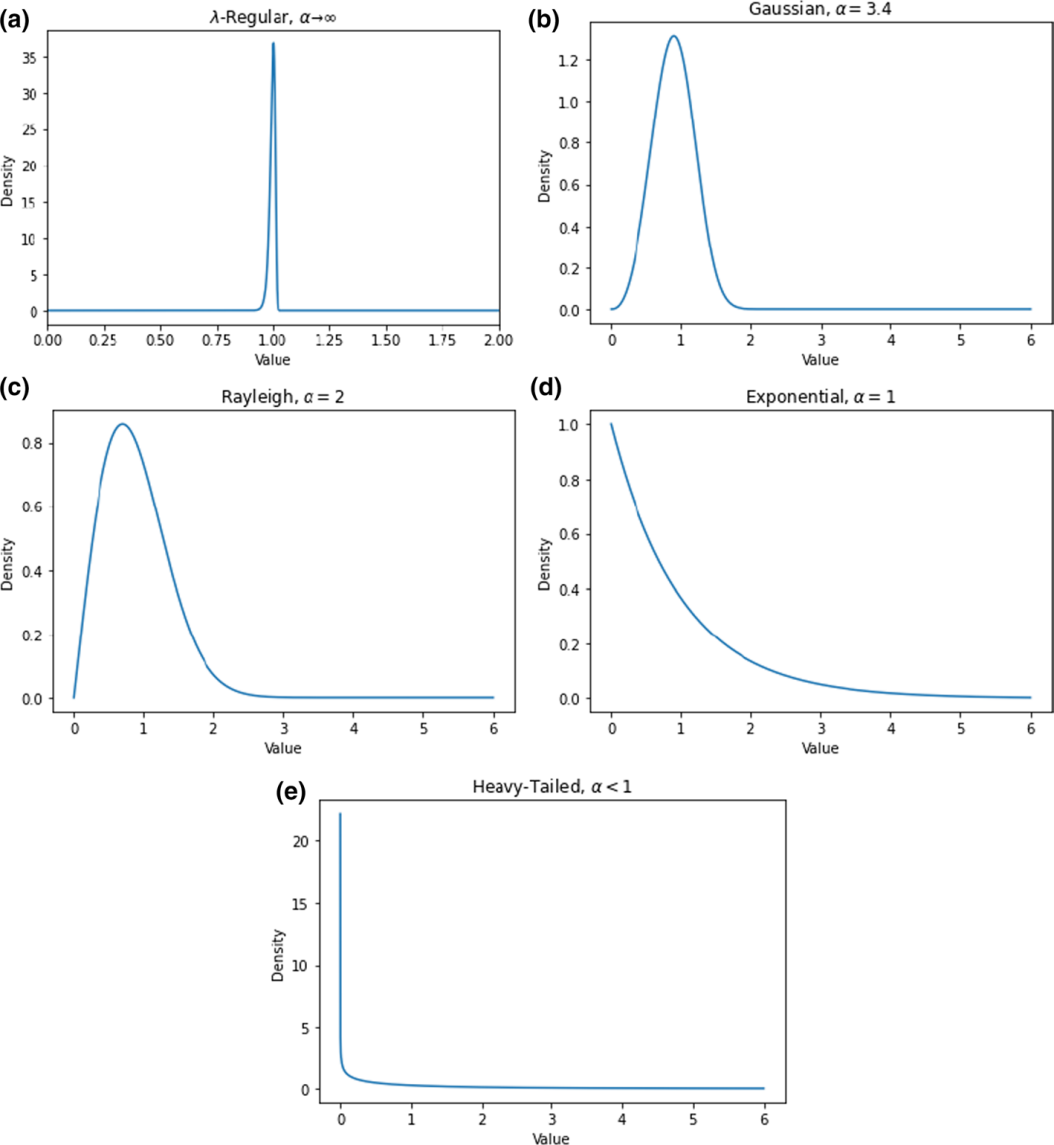
Various cases of the probability density functions of the Weibull distribution generated by varying shape parameter $\alpha$ and scale parameter $\lambda = 1$. **a** $\alpha \rightarrow \infty$ produces a nearly singular distribution at $\lambda$. **b** $\alpha =3.4$ produces a Gaussian-like distribution. **c** $\alpha =2$ produces a Rayleigh distribution. **d** $\alpha =1$ produces an exponential distribution. **e** $\alpha < 1$ produces a heavy-tailed distribution

This can be seen graphically in Fig. 1.

**Fig. 2:**
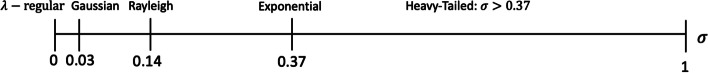
Number line for heterogeneity that highlights the special cases corresponding to canonical distributions

We can further map the whole range of heterogeneity given by $\alpha$ to the unit interval using a simple transformation. We introduce a parameter $\sigma$. This parameter is given by:

2 $$\begin{aligned} \sigma \equiv exp(-\alpha ) \end{aligned}$$

where $\alpha$ is the shape parameter of the Weibull distribution. Since the shape parameter $\alpha$ is always a positive definite real number, under the exponential transformation this $\sigma$ parameter always lies in the range (0, 1). When $\sigma$ is close to 0, the graph is homogeneous (more regular) in its degree distribution, and when $\sigma$ is close to 1 the degree distribution is heterogeneous (more heavy-tailed). We can see the special cases in terms of a number line of $\sigma$ in Fig. 2.

The resulting exponential transformation of the shape parameter results in the following modified 2-parameter Weibull probability density function:

3 $$\begin{aligned} f(x; \lambda , \sigma ) = \frac{-ln(\sigma )}{\lambda }\left( \frac{x}{\lambda }\right) ^{-ln(\sigma ) - 1} e^{-(x/\lambda )^{-ln(\sigma )}}; x\ge 0; \sigma \in (0,1) ; \lambda > \mathbb {R}^+ \end{aligned}$$

After we specify the desired level of heterogeneity in the graph, generating a degree distribution with that level of heterogeneity is simple. Two more parameters that do not relate to the heterogeneity of the graph must first be specified. These are *N*, which is the size of the graph in terms of number of nodes, and the scale parameter $\lambda$, which is the value at which the degree distribution will be centered at. These will vary from application to application. The values of these parameters will typically be estimated empirically. For instance, the size of the graph might be large if the modeller is considering a network the size of an entire country or small if instead a small neighborhood is being modelled. Similarly for the scale parameter, considering for instance social media networks, there might be data on the average number of friends users typically have.

Now with all of the parameters in hand, the following simple procedure generates a graph that has the desired heterogeneity:

1. Choose values for $\sigma$ (heterogeneity), $\lambda$ (center of degree distribution), and *N* (number of nodes).
2. Draw *N* random samples from the modified Weibull distribution specified in (3) using $\sigma ,\lambda , N$. This distribution of *N* values defines the degree distribution for the graph being generated.
3. Round each of the *N* samples to the nearest integer (since the degree of a node can only take on integer values).
4. With the rounded degree distribution from the previous step, now use the configuration model method (Newman 2018) (which samples from the space of all possible graphs corresponding to a particular degree distribution) to generate a graph.

This yields a valid graph $G(\sigma , \lambda , N)$ with the desired amount of heterogeneity as specified by $\sigma$.

Briefly, the configuration model algorithm for generating graphs proceeds as follows. Supposing that a degree $k_i$ is specified (or in this case, randomly sampled from the modified Weibull distribution), each node *i* (where $i = 1,\ldots ,N$) is given $k_i$ stubs of edges. Then, two stubs are chosen uniformly at random from the set of all stubs, thus forming an edge that connects the two nodes to which these stubs respectively belong. This process is repeated until no unmatched stubs are left. This method requires that there be an even number of stubs (i.e. that the sum of the degrees of all nodes is even). Further conditions and implementation details of the configuration method are detailed at length by Newman (2018).

## A numerical example in epidemiology

We now present a simple numerical example from epidemiology which demonstrates the utility of the heterogeneity parameter $\sigma$.

Epidemics are commonly modeled using compartmental models, such as the SIR model. In the SIR model, at any point in time, individuals are in one of three distinct states of health: susceptible, infected, or recovered. Here we will assume that infected individuals transmit the disease to susceptible individuals based on a transmission parameter ($\tau$) and recover from illness based on a recovery parameter ($\gamma$).

One of the key quantities of interest in the study of the spread of infectious diseases is the herd immunity threshold (HIT). This is the fraction of the population that has been infected by the time the number of infections peak (which occurs when $\frac{dI}{dt}=0$):

4 $$\begin{aligned} HIT \equiv \frac{I+R}{N}|_{\begin{array}{c} \frac{dI}{dt}=0 \end{array}} \end{aligned}$$

where *I* and *R* are the total number of people in the population in the infected and the recovered state respectively, and *N* is the total population size. It measures the severity of the epidemic at the peak. It has been observed that the herd immunity threshold in the context of epidemic simulations on graph networks, are quite sensitive to the amount of heterogeneity present in the contact network between people, which is represented by a graph.

Below, we present the results of SIR simulations of epidemics run on graphs with differing levels of heterogeneity, as given by $\sigma$. The method for running the SIR epidemic on a network is given by the following simulation procedure:

Given a graph $G(\sigma ,\lambda ,N)$ and the epidemic parameters for transmission probability ($\tau$) and recovery probability ($\gamma$):

1. At time $t_0$, fix a small fraction $\epsilon$ of nodes to be chosen uniformly on the graph and assign them to the Infected state. The remaining $(1 - \epsilon )$ fraction of nodes start as Susceptible.
2. At each time step $i \in [1,T]$, given a pair of nodes (one susceptible, the other infected) connected by an edge, the susceptible node becomes infected with probability $\tau$.
3. At each time step $i \in [1,T]$, each infected node recovers with probability $\gamma$.
4. At time step *T*, record the herd immunity threshold (HIT) at the peak of the epidemic.
5. Repeat steps (1–4) n = 150 times for each value of $\tau$.
6. Repeat steps (1–5) for each value of $\sigma$.

**Fig. 3:**
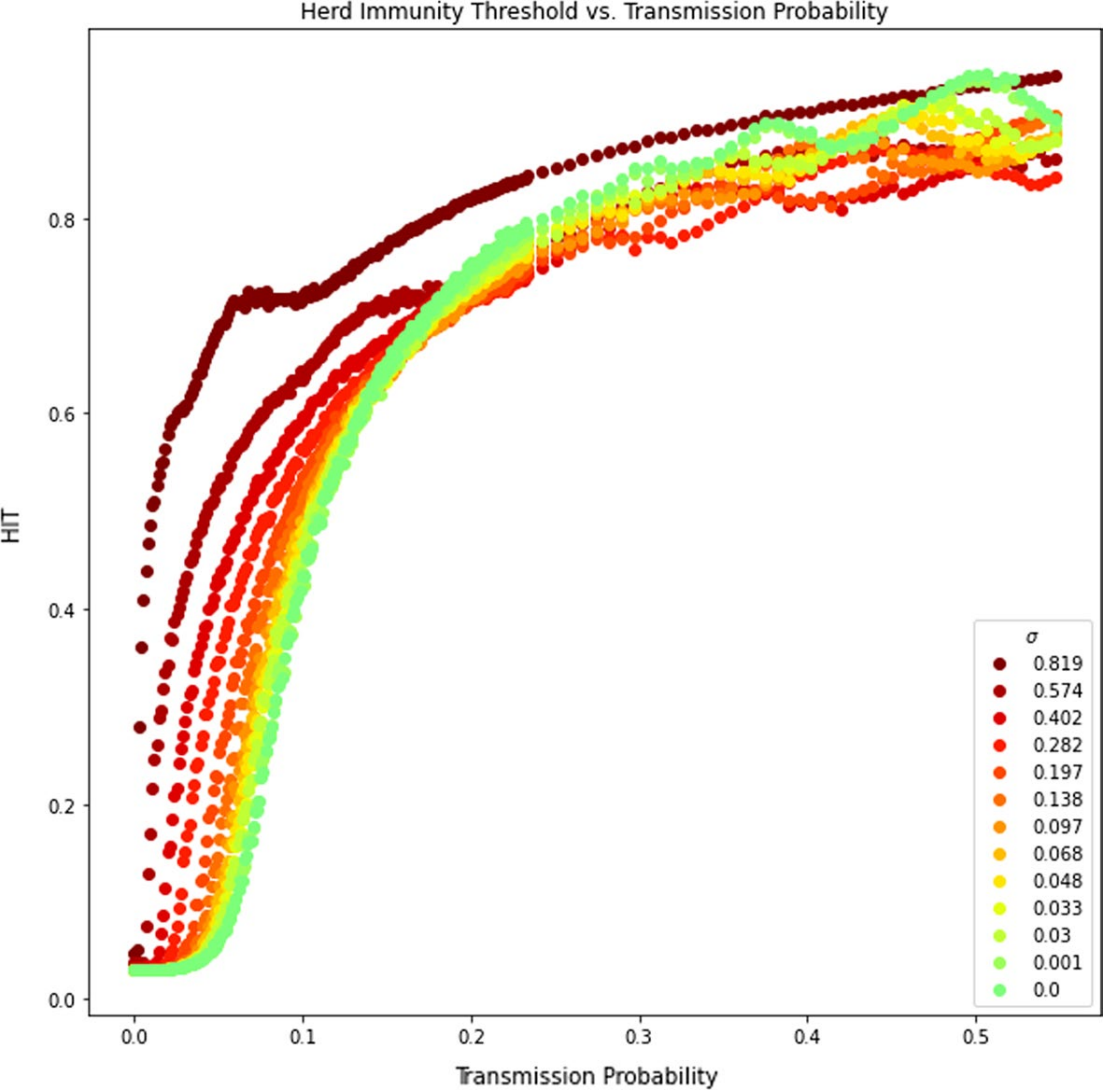
The herd immunity threshold for SIR epidemic simulations on networks with varying levels of heterogeneity ($\sigma$) as a function of transmission probability. $\lambda = 5, N = 1000$. Points represent the average of n = 150 simulation runs

The results are shown in Fig. 3. This sensitivity analysis highlights two features that vary with heterogeneity. First, the red curves, with higher heterogeneity, lack a sub-critical regime that the more homogeneous curves have. This is recapitulates well-known behavior described in scale-free networks (Pastor-Satorras and Vespignani 2001). Second, the HIT is not monotonic in the transmission probability for all graph types. Meanwhile, classical intuition suggests the proportion of graph reached at peak infections to be higher on average as the transmission probability increases. This novel counter-intuitive behavior is explored in depth by Ozbay et al. (2022).

This unexpected behavior was easily uncovered with a sensitivity analysis of this parameterization of heterogeneity. Because different graphs of interest are special cases corresponding to specific values of $\sigma$, we can easily interpolate between many different graphs and do so in a way that is precise and quantitative. Because the effect of heterogeneity can be completely isolated using this modified Weibull method, the observed differences in the realizations of the HIT can entirely be attributed to differences in degree distribution heterogeneity. That several conclusions regarding the impact of network heterogeneity could be quickly elucidated from inspection of Fig. 3 highlights the usefulness of the modified Weibull distribution in capturing dependencies of variables of interest (in this case HIT) on network structure.

## Analytical calculations using the degree distribution

Using the probability density function given by (3), the moments of the degree distribution can be expressed analytically For example, the mean ($<k>$) and variance ($<k^2>$) of the degree distribution are as follows:

5 $$\begin{aligned} <k>&= \lambda \Gamma \left( \frac{-1}{ln\sigma }+1\right) \end{aligned}$$

6 $$\begin{aligned} <k^2>&= \lambda ^2\left( \Gamma \left( \frac{-2}{ln\sigma }+1\right) -\left( \Gamma \left( \frac{-1}{ln\sigma }+1\right) \right) ^2\right) \end{aligned}$$

where $\Gamma$ denotes the gamma function. These expressions are derived directly by computing the first and second moments of the modified Weibull distribution presented above (Eq. 3). Thus depending on the particular problem, one could in principle make theoretical predictions of the impact of heterogeneity using the standard techniques of calculus. This could obviate the necessity for performing the potentially computationally costly calculation of quantities that are explicit functions of the degree distribution’s moments.

To illustrate this conceptually, we first note that there are closed form equations for a number of quantities associated with the configuration model expressed in terms of the degree distribution (Newman 2018). Examples of such calculations include the following: the clustering coefficient, the existence of the giant component, the mean size of a component of a randomly chosen node, the critical occupation probability for the giant percolation cluster, and the expected largest eigenvalue.

As an example, we will characterize the sensitivity of the largest eigenvalue of a graph to changing graph heterogeneity. In the large network limit, the theoretical prediction for the largest eigenvalue in the configuration model ($\kappa _{1,theory}$) is given by the ratio of the variance to the mean of the degree distribution (see Equation 17.94 in Newman (2018), p. 700). Using (5) and (6) gives the following prediction:

7 $$\begin{aligned} \kappa _{1,theory}=\frac{<k^2>}{<k>} = \lambda \left[ \frac{\Gamma (\frac{-2}{ln\sigma }+1)-(\Gamma (\frac{-1}{ln\sigma }+1))^2}{\Gamma (\frac{-1}{ln\sigma }+1)}\right] \end{aligned}$$

**Fig. 4:**
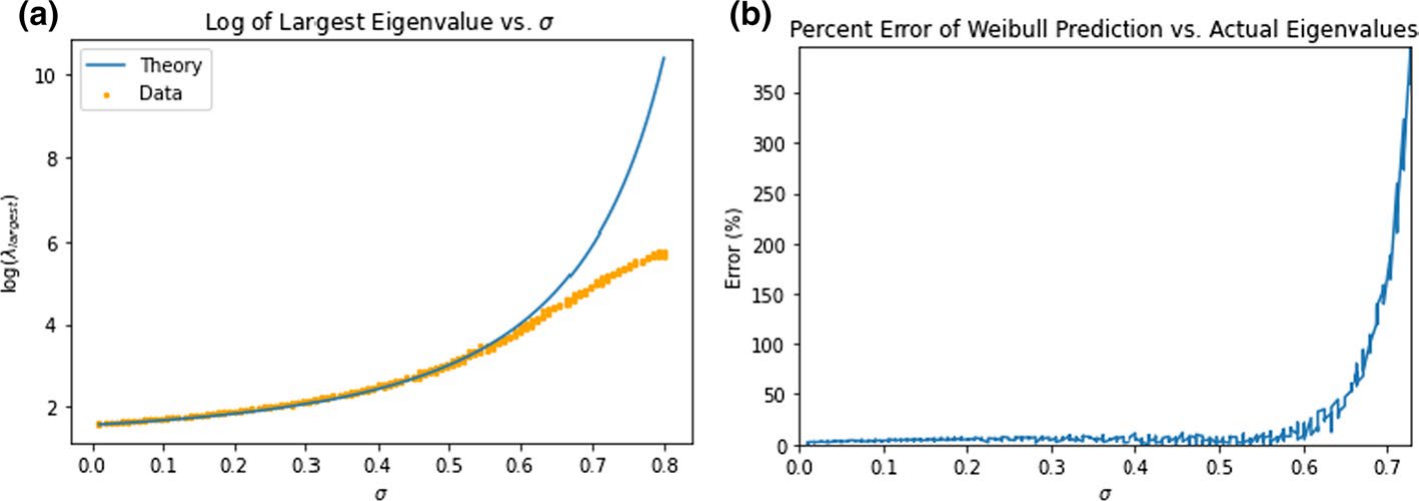
**a** Comparison of the logarithms of the largest eigenvalue calculated by the theory of (Newman 2018) and the empirically calculated value as a function of heterogeneity, $\sigma$ for system size N = 5000. **b** Percent absolute error between predicted eigenvalue and empirically calculated eigenvalue, for various values of $\sigma$

Figure 4 shows both the theoretical prediction of the largest eigenvalue given by Eq. (7) and the ground truth calculated empirically from generated graphs of the same parameter values $G(\sigma , \lambda , N)$. We see that the largest eigenvalue of the graph increases monotonically with the heterogeneity of the graph. We see towards the homogeneous limit (i.e. when $\sigma$ is small), the theory and data agree remarkably well. As heterogeneity increases, the discrepancy between the predicted eigenvalue and the ground truth grows quickly, indicating a breakdown of the theory in the regime of extremely heavy-tailed graphs. This breakdown can potentially be explained by the fracturing of the graph into many components in the very heavy-tailed regime, as discussed further in the Discussion. Ultimately, we emphasis the ability of our method to produce meaningful predictions solely in terms of the parameterization of the degree distribution.

## Discussion

Graphs are expressive modelling tools that can be, by the same token, complicated and difficult to characterize quantitatively in all of their features. In particular, one of the key advantages of graphs is that they can capture highly local information and interactions in a system. This makes the heterogeneity of the degree distribution a quantity of great interest for various applications of graphs and dynamical processes on graphs. The parameter $\sigma$ presented allows for several canonical graphs to be considered as special cases and easy interpolation between them. As the examples showed, processes on graphs and global statistics can be sensitive to local heterogeneity, necessitating a quantitative formulation of graph heterogeneity for those problems.

The key to the method proposed here is the choice of the Weibull distribution as a basis for deriving the different graphs. While other parameterizations using alternative statistical distributions are certainly possible and have been previously proposed as discussed in the literature review, we justified the use of the Weibull distribution on the following grounds. First, the Weibull distribution constitutes what can be called a *statistical*, as opposed to a *mechanistic* network interpolation scheme. Recent work in graph theory has stressed the importance of the difference between statistical and mechanistic network models (Chen et al. 2020). Whereas statistical models specify a likelihood for each instance of a graph, mechanistic network models specify a set of mechanisms by which a network grows and changes over time, and are thus harder to characterize with a likelihood. In line with this distinction, existing interpolation methods that are able to recover a large number of class of graphs of interest are mechanistic in nature. They interpolate between different classes of graphs by continuously varying the parameters that control the attachment probability for a new node in the graph generation procedure. In contrast to these methods, the Weibull method interpolates at the level of the distribution that characterizes the graph, rather than the distribution induced by a particular preferential attachment-type rule.

This presents one shortcoming and one advantage that is common when contrasting statistical and mechanistic graphs. The shortcoming is that if the network or class of network being studied is well-known to have been generated by a simple physical process, then the mechanistic model, both for generating graphs and interpolating between graphs, likely offers a better physical interpretation than a statistical network model. The advantage of using the Weibull method is that it allows the modeller to control and vary only the degree distribution, rather than other elements of the graph topology that cannot be achieved when using an attachment rule.

If, for example, a sensitivity analysis is being conducted that seeks to understand only the expected effects of degree distribution, then an interpolation scheme that can, on average, vary only and exactly the degree distribution is very useful for isolating the effect of first order (first neighbor) topology of the graph. In turn, other parameters that control higher order elements of the graph topology can be introduced and varied separately. Particularly in cases where it is less clear that a simple physical mechanism, as in preferential attachment, generated the network, the Weibull method both recovers many distributions of interest and avoids artifacts of any particular attachment rule that might remain in generating the graph. Since the configuration model is equally likely to generate any graph of the given degree distribution.

More generally, a quantitative measure or parameterization of graph heterogeneity should ideally possess two qualities: expressiveness and interpretability. By expressiveness, we mean the parameterization should be broad enough to incorporate many of the cases of interest. There are many canonical graphs, so ideally a measure of heterogeneity should be able to assign a value to a reasonable number of them. One way to incorporate more possible graphs is to construct a more complex statistical distribution. There is a whole zoology of different statistical distributions. And with enough parameters one could construct a procedure that could generate arbitrary numbers of different graphs. However, this increasing complexity in parameterization comes at the cost of interpretability. Ideally it would be preferable to have a parameterization using a single scalar value as the effect of variation in that scenario is easy to interpret. One can make straightforward apples-to-apples comparisons between graphs in such a situation. In contrast, the interpretation of comparing two graphs when the parameterization is vector-valued is more complicated. Clearly there is a trade-off that occurs between expressiveness and interpretability, and a balance must be struck. In the context of a control parameter for heterogeneity, the modified Weibull distribution with its parameter $\sigma$ provides a balanced trade-off in terms of interpretability and expressiveness. As demonstrated above, many graphs of interest are captured strictly in terms of a single heterogeneity parameter.

**Fig. 5:**
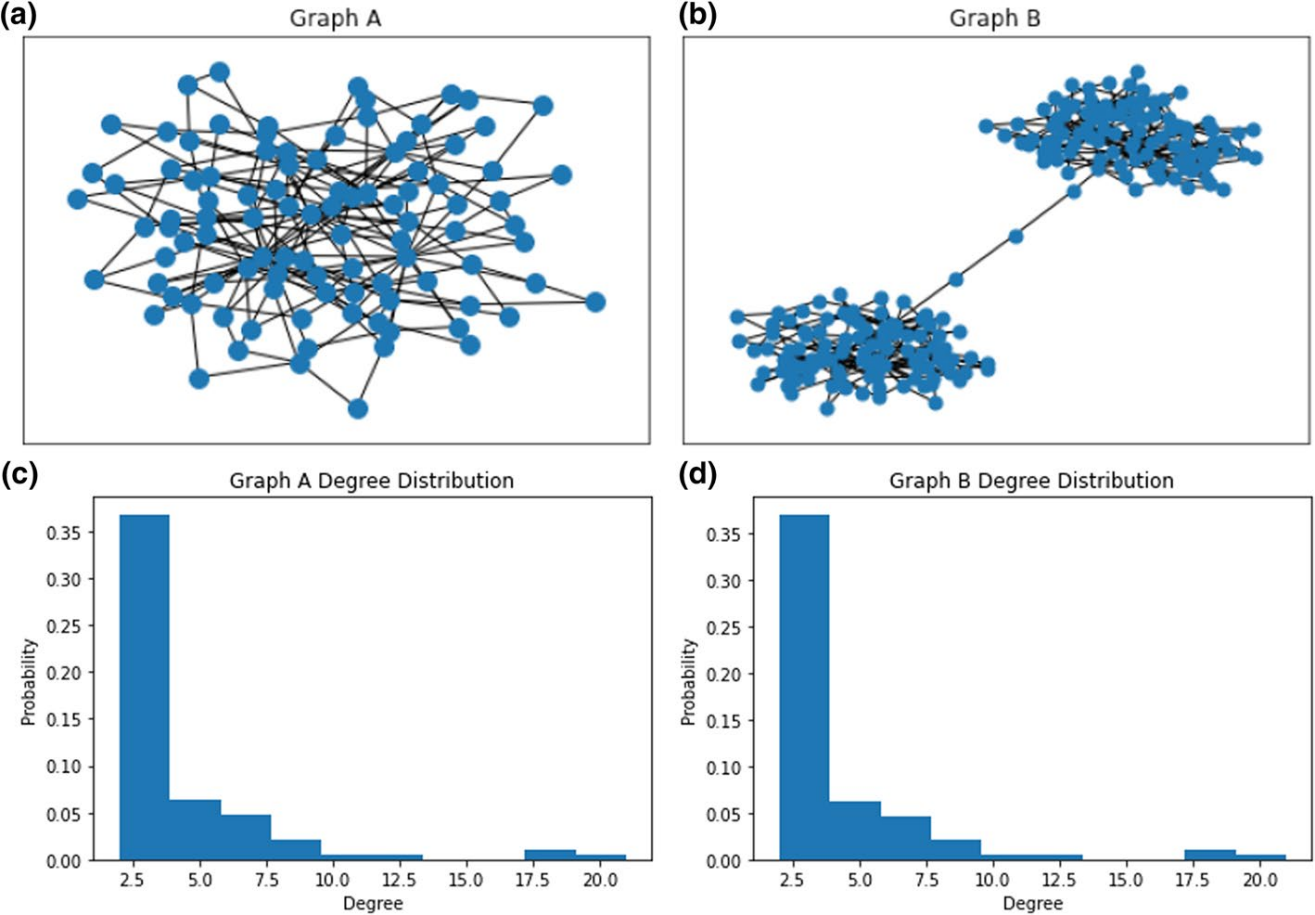
**a** Visualization of a Albert–Barabási graph with 100 nodes total and 2 nodes preferentially attached to each node. **b** A barbell graph constructed by creating a copy of Graph A and attaching the two copies via a path graph. **c** Histograms of Graph A degree distribution. **d** Histogram of Graph B degree distribution

There are two main limitations of the stated parameterization of network heterogeneity. First, its primary assumption, that the degree distribution of a graph will generally provide a complete picture of its overall topological heterogeneity, is a potential limitation. This assumption does not always hold true. In particular, the degree distribution only determines first-order and thus highly local topological properties about the graph. As can be seen in Fig. 5, we provide an example of two graphs with nearly identical degree distributions that are substantially different in their global topological features. In the example given, this manifests as Graph A lacking any protrusions or visible modularity, while Graph B has two distinct modules joined in a “barbell”-like shape. By focusing only on the degree distribution, our graph generation procedure is decoupled to the presence or absence of these large-scale features. Clearly though, such differences in global topological heterogeneity can have meaningful effects on stochastic processes on graphs. Despite their global structure being very different, both Graph A and B are in the space of graphs that can be generated by the configuration model, which only requires the degree distribution as input. While it is possible that Graph B could be constructed by the configuration model in principle, in practice Graph B has a very low probability of being constructed under this (or any random graph) construction procedure. That is because, for a given degree distribution, whether or not any two nodes are connected is completely random under the configuration model. Thus with a sufficiently large enough network, in the process of randomly connecting nodes it becomes extremely unlikely that roughly half of the nodes would not share any connections with the other half of the nodes. Therefore, graphs with some level of modularity make up a vanishingly small proportion of the space of graphs that are generated by this procedure. In contrast, real world networks can display substantial modularity which might be lost by only characterizing a degree distribution. Future work may look for a generalization of this approach beyond the degree distribution or configuration model.

Second, the method proposed may not always yield a fully connected network in the heavy-tailed regime. While this method always generates a network assuming the sum of the degrees of all nodes is even, it does not guarantee a fully connected graph. Indeed, for extremely heavy-tailed graphs, we find a significant fraction of nodes may be disconnected from the largest component. This is a known feature of the configuration model. Depending on the system being studied, this can be partially remedied by studying very large networks or if the analysis of a giant component is sufficient. Although this presents a fundamental limitation on graph construction in the heavy-tailed regime, we believe it is a necessary trade-off to have more control over the degree distribution, which allows for precise analysis regarding the effect of heterogeneity.

In conclusion, the parameter $\sigma$ presented a simple means of controlling heterogeneity in a graph quantitatively which, in turn, can be used to better and more precisely understand the effect of changing graph topology on processes involving graphs.

## Data Availability

The code and datasets used and/or analysed during the current study are available from the corresponding author on reasonable request.
